# William Lawrence

**Published:** 1857-06

**Authors:** 


*William Lawrence.*—This celebrated writer and surgeon, according to the author of the Clinical Reports, published in the *London Lancet*, "is as active and expert in his operations, even those of the most delicate character, at the present time, as in his earlier days." Mr. L. is now over seventy years of age, and we can scarcely credit the above, for we saw him perform many operations during the winter of 1846–7, and were even then often reminded of that saying of Horace: *Multa se nem circumveniunt incommoda.* Mr. Lawrence is probably the most learned surgeon that Great Britain has ever produced.—*Western Lancet.*

